# Metformin alleviates irradiation-induced intestinal injury by activation of FXR in intestinal epithelia

**DOI:** 10.3389/fmicb.2022.932294

**Published:** 2022-10-13

**Authors:** Jing-Yu Yang, Meng-Jie Liu, Lin Lv, Jin-Rong Guo, Kai-Yue He, Hong Zhang, Ke-Ke Wang, Cui-Yun Cui, Bei-Zhan Yan, Dan-Dan Du, Jin-Hua Wang, Qiang Ding, Guo-Long Liu, Zhi-Xiang Xu, Yong-Ping Jian

**Affiliations:** ^1^School of Life Sciences, Henan University, Kaifeng, China; ^2^Department of Medical Oncology, Guangzhou First People's Hospital, School of Medicine, South China University of Technology, Guangzhou, China; ^3^Jiangsu Cancer Hospital, Jiangsu Institute of Cancer Research, The Affiliated Cancer Hospital of Nanjing Medical University, Nanjing, China; ^4^Department of Blood Transfusion, Henan Provincial People's Hospital, People's Hospital of Zhengzhou University, Zhengzhou, China; ^5^Department of Internal Medicine, Ningjin County People's Hospital, Dezhou, China; ^6^Department of Medicine, University of Alabama at Birmingham, Birmingham, AL, United States

**Keywords:** irradiation-induced intestinal injury, microbiota, *Lactobacillus*, metformin, intestinal barrier, farnesoid X receptor (FXR)

## Abstract

Abdominal irradiation (IR) destroys the intestinal mucosal barrier, leading to severe intestinal infection. There is an urgent need to find safe and effective treatments to reduce IR-induced intestinal injury. In this study, we reported that metformin protected mice from abdominal IR-induced intestinal injury by improving the composition and diversity of intestinal flora. The elimination of intestinal microbiota (Abx) abrogated the protective effects of metformin on irradiated mice. We further characterized that treatment of metformin increased the murine intestinal abundance of *Lactobacillus*, which mediated the radioprotective effect. The administration of *Lactobacillus* or fecal microbiota transplantation (FMT) into Abx mice considerably lessened IR-induced intestinal damage and restored the radioprotective function of metformin in Abx mice. In addition, applying the murine intestinal organoid model, we demonstrated that IR inhibited the formation of intestinal organoids, and metformin alone bore no protective effect on organoids after IR. However, a combination of metformin and *Lactobacillus* or *Lactobacillus* alone displayed a strong radioprotection on the organoid formation. We demonstrated that metformin/*Lactobacillus* activated the farnesoid X receptor (FXR) signaling in intestinal epithelial cells and hence upregulated tight junction proteins and mucins in intestinal epithelia, increased the number of goblet cells, and augmented the mucus layer thickness to maintain the integrity of intestinal epithelial barrier, which eventually contributed to reduced radiation intestinal injury. In addition, we found that *Lactobacillus* abundance was significantly increased in the intestine of patients receiving metformin while undergoing abdominal radiotherapy and the abundance was negatively correlated with the diarrhea duration of patients. In conclusion, our results demonstrate that metformin possesses a protective effect on IR-induced intestinal injury by upregulating the abundance of *Lactobacillus* in the intestine.

## Background

Radiotherapy is an important adjuvant treatment for abdominal tumors (Miller et al., 2019). Intestinal radiotoxicity is a major limitation to the application of radiotherapy in abdominal tumors although strategies for the therapy have been greatly improved (Fransson and Widmark, 2007; De Ruysscher et al., 2019). Irradiation (IR)-induced intestinal injury destroys the intestinal mucosal barrier (Andreyev, 2005; Hauer-Jensen et al., 2014), leading to severe intestinal infection (Chaves-Pérez et al., 2019; Jian et al., 2021). Therefore, there is an urgent need to find safe and effective treatments to reduce IR-induced intestinal injury (Hauer-Jensen et al., 2014).

Human gastrointestinal microbiota, also known as gut microbiota, comprises microorganisms that live in the human digestive tract. It plays a critical role in the maintenance of nutrient absorption, metabolism, and immune function (McKenzie et al., 2017). It was reported that intestinal flora protects germ-free mice from radiation-induced intestinal damage and death (Guo et al., 2020), suggesting that intestinal dysbiosis plays a crucial role in the pathogenesis of IR-induced intestinal injury. Gerassy-Vainberg et al. (2018) reported rectal radiation-induced dysbiosis, hence increasing intestinal inflammation susceptibility. However, it remains unclear whether alterations in gut microbiota affect the occurrence and outcome of radiation-induced intestinal damage. Therefore, elucidating the mechanism of interaction between the microbiome and the host may provide potential therapeutic benefits for patients with radiation-induced intestinal injury.

We previously found that *Lactobacillus* activates FXR–FGF15 signaling in intestinal epithelial cells and hence promotes DNA damage repair (Jian et al., 2022). FXR is a bile acid-responsive transcriptional regulator for liver bile metabolism. The expression and function of FXR in the intestine (especially the ileum), immune cells, and endothelial tissues have recently been characterized (Gadaleta et al., 2011; Verbeke et al., 2014). FXR knockout mice not only show obvious liver inflammation and fibrosis but also develop an inflammatory bowel disease-like phenotype, with increased intestinal inflammation and permeability (Wang et al., 2008). FXR activation promotes the expression of tight junction proteins to improve the ileal barrier function (Verbeke et al., 2015), demonstrating a crucially protective role of FXR in the intestine.

Metformin, a first-line agent for the treatment of type 2 diabetes, improves upper small intestine microbiota composition and restores the sodium-glucose cotransporter-1 (SGLT1)-dependent glucose sensing to regulate glucose homeostasis (Wu et al., 2017; Bauer et al., 2018). Metformin was previously reported to enhance radiation sensitivity (Gulati et al., 2020) and improve radiation-induced lung injury, alleviate pulmonary fibrosis and inflammatory infiltration, and hence bear a potential for application in radiation protection (Wang et al., 2017). Chen et al. reported metformin alleviated radiation-induced intestinal injury by optimizing mitophagy, an AMPK-dependent process (Chen et al., 2020). The effect of metformin on the gut microbiota in radiation-induced intestinal injury is unclear. In this study, we established an animal model of abdominal IR-induced intestinal injury and characterized the role and underlying signaling of a specific microorganism, *Lactobacillus*, and its activator, metformin, in the prevention and treatment of radiation-induced intestinal injury in mice. Our results suggest that corrections of abnormal gut microbiota, such as administration of *Lactobacillus* or elevation of the probiotic by metformin, protected IR mice and abdominal IR patients from intestinal injury. Our findings reveal a novel target for preventing radiation-induced intestinal damage.

## Materials and methods

### Establishment of radiation injury model

Female BALB/c mice were maintained in a specific pathogen-free (SPF) animal facility at Henan University. Mice were kept on a 12-h light-dark cycle with food and water available *ad libitum*. Metformin (250 mg/kg/day) was administered intragastrically for 7 days before and 3 days after abdominal IR (Higurashi et al., 2016). In the model, 8 Gy one-time abdominal IR with a dose rate of 1.0 Gy/min by X-ray radiation source was used (Ottewell et al., 2003). After radiation, mice were housed in an animal facility for routine observation and treatment. Ethical regulations for animal testing and research at Henan University were strictly implemented. Protocols for animal usage were approved by the institutional animal care and use committee (IACUC) at Henan University, China.

### Collection of murine feces

Feces of mice were collected after IR. For 16S rRNA sequencing, 30 mg of feces were kept in a sterilized Eppendorf tube.

### Collection of ileal contents

After the mouse was euthanized, the ileocecal junction of the intestine was identified and located. The ileum was connected to one end of the ileocecal junction. Within 5 cm of the distal ileum, 60 mg of ileal content was collected and stored in sterilized Eppendorf tubes at −80°C.

### 16S rRNA sequencing

Both mucosa and the epithelial tissue in the ileum of mice were scraped for collecting ileal contents. DNA from the ileal contents of mice was extracted for PCR amplification. The amplified sample was confirmed by 2% agarose gel electrophoresis. The high-throughput sequencing involved was completed by Shanghai Meiji Biology Company (Shanghai, China) (Jian et al., 2022).

### Immunohistochemistry

The paraffin sections were dewaxed and hydrated for thermal antigen retrieval, endogenous peroxidase blocking, antibody action, and development as we reported previously (Jian et al., 2022). The immunoreaction was quantified using the immunohistochemistry (IHC) plugin of the Image J software (Jian et al., 2022).

### Hematoxylin-eosin staining and histological injury score of the intestine

The preparation of paraffin sections and hematoxylin-eosin (H&E) staining of the intestine were performed as we reported previously (Jian et al., 2022). Intestinal damage was scored and quantitated in the H&E staining. The intestinal mucosal injury was classified using Chiu's method (Chiu et al., 1970) as follows: 0, intestinal mucosal villus without abnormality; 1, cystic gaps and capillary hyperemia under the epithelium in villus apex; 2, cystic gaps enlarged under the epithelium, edema expanded to lamina propria, and central cheliferous vessels dilated; 3, severe edema in lamina propria, deterioration, and necrosis of intestinal epithelial cells (IECs), and abscission of a small number of villus apex; 4, open of lamina propria, dilation of capillary, and hyperemia; degeneration, necrosis, and exfoliation of IECs, abscission of a number of villi; and 5, fragmentation of lamina propria, and bleeding or ulceration, abscission of the complete villus (Mori et al., 2011).

### Electron microscopy

The ilea of mice were isolated, rinsed with 0.9% saline, and prefixed with 4% glutaraldehyde. Samples were then processed to complete TEM biological sample preparation and electron microscopy observation (Ding et al., 2020).

### Fecal microbiota transplantation

Fresh fecal pellets were collected in PBS with a concentration of 50 mg feces/ml. Pooled samples were centrifuged and the supernatant was used for fecal microbiota transplantation (FMT) (van den Berg et al., 2021). A total of 200 μl of FMT or PBS alone were intragastrically administered into microbiota-eliminated mice each day for 4 weeks continuously before and 1 week after IR to reconstitute the intestinal microbiome of mice (Jian et al., 2022).

### Intestinal organoid culture

Mice were euthanized with carbon dioxide, and intestinal crypts were isolated. Villi of the intestine were scraped off with a coverslip and incubated in PBS and 2 mM EDTA at 4°C for 20 min, and then incubated at 37°C for 8 min. The digested crypts were then centrifuged at 300 *g* for 3 min, embedded in Matrigel, polymerized at 37°C for 30 min, and supplemented with a complete culture medium (Sato et al., 2011; Sato and Clevers, 2013).

### Treatment of intestinal organoids with *Lactobacillus plantarum*

Intestinal organoids were seeded in a 96-well plate with 10% Matrigel and exposed to *Lactobacillus plantarum* (*LP*) with a multiplicity of infection (MOI) of 100 for 4 h. Wells containing organoids were then washed with the medium containing the bacteria and then removed. Culture wells were washed 3 times with a medium containing 1% penicillin-streptomycin and 40 μg/ml gentamicin. Organoids were then placed in the medium with the antibiotics aforementioned for up to 3 days and measured to determine the organoid size (Yang et al., 2022).

### Intestinal organoid size

Intestinal organoids were seeded and cultured in a 96-well plate with 10% Matrigel. The growth of intestinal organoids was assessed with a 10× objective light microscope (day 1). Intestinal organoids were then exposed to treatments for another 48 h. Images of intestinal organoids were captured using the EVOS FL cell imaging system at the end of the 3rd day (Thermo Fisher Scientific) (Park et al., 2021). Intestinal organoid sizes were determined using the Image J software.

### Intestinal organoid viability

Intestinal organoids were prepared and cultured as aforementioned. MTS assay buffer (10 μl) (CellTiter 96 AQueous One Solution, Promega, Madison, WI, USA) was added to each well containing 100 μl of basal medium 3 h before the end of the experiment. After incubation, the optical density at 490 nm was measured using a BioTek microplate reader (BioTek Instruments Inc., Winooski, VT, USA) (Park et al., 2021).

### Establishment of intestinal microbiota-eliminated mice

Specific pathogen-free mice were treated with a mixture of 4 antibiotics including 200 mg/L ampicillin, 200 mg/L metronidazole, 200 mg/L neomycin, and 100 mg/L vancomycin in drinking water for 4 weeks (Castellanos et al., 2018; Scott et al., 2018; Zarrinpar et al., 2018; Yang et al., 2019). All antibiotics were purchased from Meilunbio, China. At the end of the treatment, the intestinal content of mice was collected for 16S rRNA sequencing to validate the elimination of intestinal microbiota.

### Application of *Lactobacillus* in mice

*Lactobacillus plantarum (LP)* was obtained from the American Type Culture Collection (202195) and cultured in *Lactobacillus* MRS broth (Panigrahi et al., 2017). To reconstitute the intestinal microbiome of mice before IR, 100 million CFU of *LP* in 0.2 ml of PBS or PBS alone was intragastrically administered into microbiota-eliminated mice each day for 4 weeks.

### Periodic acid-Schiff staining

Paraffin sections were dewaxed and hydrated as described previously. The slide was stained with 1% periodic acid for 10 min and washed 3 times with distilled water for 5 min each time. The slide was then stained with Schiff reagent for 30 min. Differentiation, development, nucleus staining, dehydration, transparency, and sealing were carried out following the protocol reported previously (Hänninen et al., 2018).

### Collection of stool from patients with cervical cancer

We collected feces from cervical cancer patients registered in the department of gynecology of Jiangsu Cancer Hospital and undergoing abdominal radiotherapy. The irradiation dose was 2 Gy/time, 5 times/week for 5 consecutive weeks. The inclusion criteria were as follows: (1) 30–60 years of age; (2) receiving abdominal radiation therapy; (3) with or without clinical manifestations of radiation-induced intestine injury (tenesmus, diarrhea, rectal bleeding, mucus and fecal incontinence, etc.); (4) with or without metformin administration recently. The exclusion criteria were as follows: radiotherapy was interrupted, inflammatory bowel disease, and tumor involving the compressing bowel. Screened according to the inclusion criteria, fecal samples from 45 female patients were collected for 16S rRNA sequencing. In addition, 15 healthy female subjects, aged between 30 and 60, were recruited locally, and their stool samples were collected to serve as normal controls.

### Statistical analysis

Experimental data were analyzed using SPSS 17.0. Student's *t*-test and one-way ANOVA were used for the significant test. A *P* < 0.05 (^*^ *P* < 0.05, ^**^ *P* < 0.01, ^***^ *P* < 0.001) was set to be statistically significant, whereas when the *P* > 0.05, the experimental results were defined as statistically insignificant.

## Results

### Metformin mitigates intestinal damage in mice after IR

**Figure 1:**
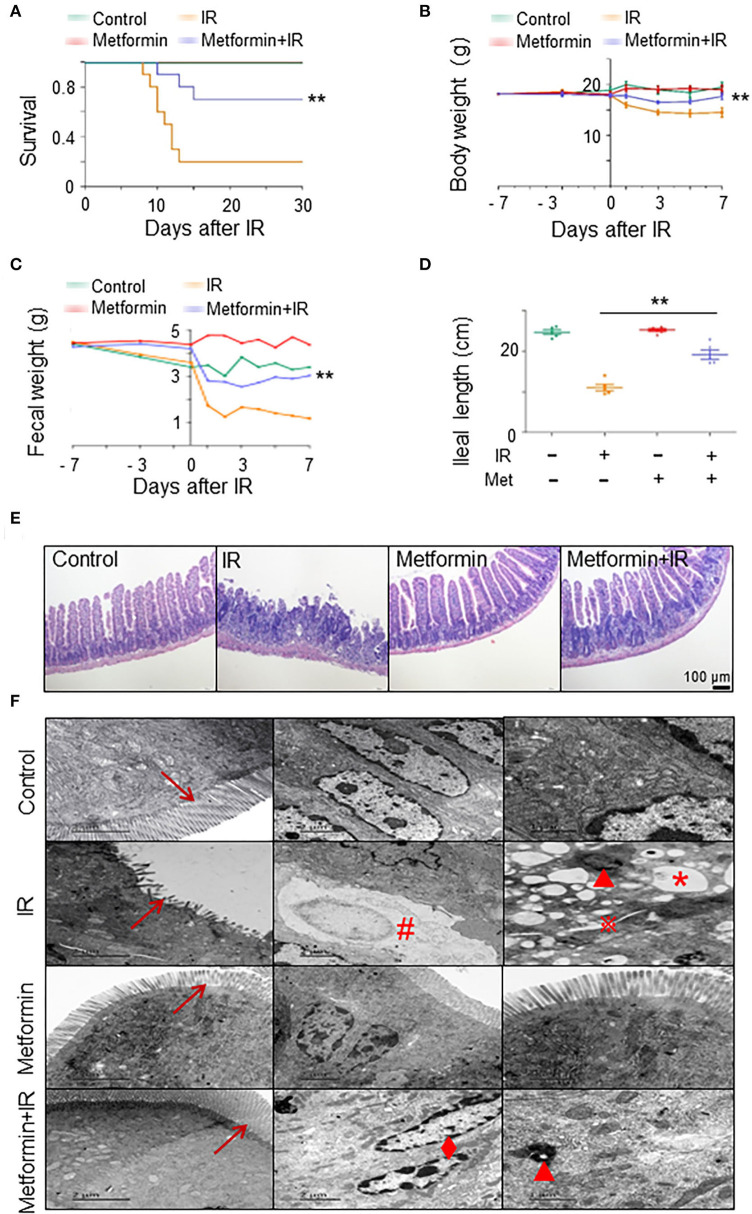
Metformin mitigates intestinal damage in IR mice. Metformin was intragastrically administered to mice at a dose of 250 mg/kg/day. Each mouse was administered continuously for 7 days before and 3 days after radiation for a total of 10 days. Mice were given 8 Gy one-time abdominal IR with a dose rate of 1.0 Gy/min in an X-ray radiation source or a mock treatment. Ilea were collected for light and electron microscopy 3 days after the IR. Mice were analyzed for **(A)** survival, **(B)** body weight, **(C)** fecal weight, **(D)** ileal length, **(E)** H&E staining of ilea, and **(F)** electron microscopic detection of ilea. → , microvillus of intestinal epithelial cells; 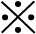, increased intercellular space; *, vacuolar degeneration; ▴, increased cytosolic lysosomes; ♦, chromatin margination; #, cell necrosis. Data represent mean ± SEM. ** *P* < 0.01, *n* = 5, compared with IR. Also see Supplementary Figure S1.

To establish a model of irradiation (IR)-induced intestinal injury, we administered abdominal IR of 8 Gy to BALB/c mice (Cui et al., 2017). To determine the radioprotective effects of metformin on IR mice, we analyzed the survival, body weight, and intestinal injury in abdominal IR mice treated with metformin. As shown in Figures 1A,B, treatment with metformin led to a marked improvement in the survival and body weight of IR mice (Figures 1A,B). Metformin did not affect the blood glucose, drinking water, and diet intake of mice in our experimental settings (Supplementary Figures S1A–C). In contrast to the striking reduction of feces and markedly decreased ileal length in IR mice, administration of metformin substantially improved fecal output and prevented the mice from IR-induced ileum shortening (Figures 1C,D). The application of metformin reduced histological injury score and abrogated IR-induced intestinal damages, such as reduction in intestinal villus height and cell death (Figure 1E; Supplementary Figure S1D). Under the electron microscope, the epithelial microvilli of control and metformin-treated mice were arranged in order, and no abnormalities in the endoplasmic reticulum, mitochondria, intercellular gap, and nuclear were displayed. In IR mice, however, epithelial microvilli were rare, short, sloughing, and irregularly arranged (Figure 1F; Supplementary Figure S1E). Increased cytosolic lysosomes, vacuolar degeneration, nuclear condensation, fragmentation, dissolution, chromatin margination, increased intercellular space, and cell necrosis were observed in intestinal epithelia of mice treated with IR (Figure 1F). Treatment of metformin led to a striking improvement in IECs of abdominal IR mice under the electron microscopy analysis, with only marginal damages observed in epithelial microvilli and chromatin margination (Figure 1F). These results demonstrate that radiation-induced intestinal tissue damages were relieved with the application of metformin in mice.

### Metformin improves the composition and abundance of intestinal flora in mice after IR

**Figure 2:**
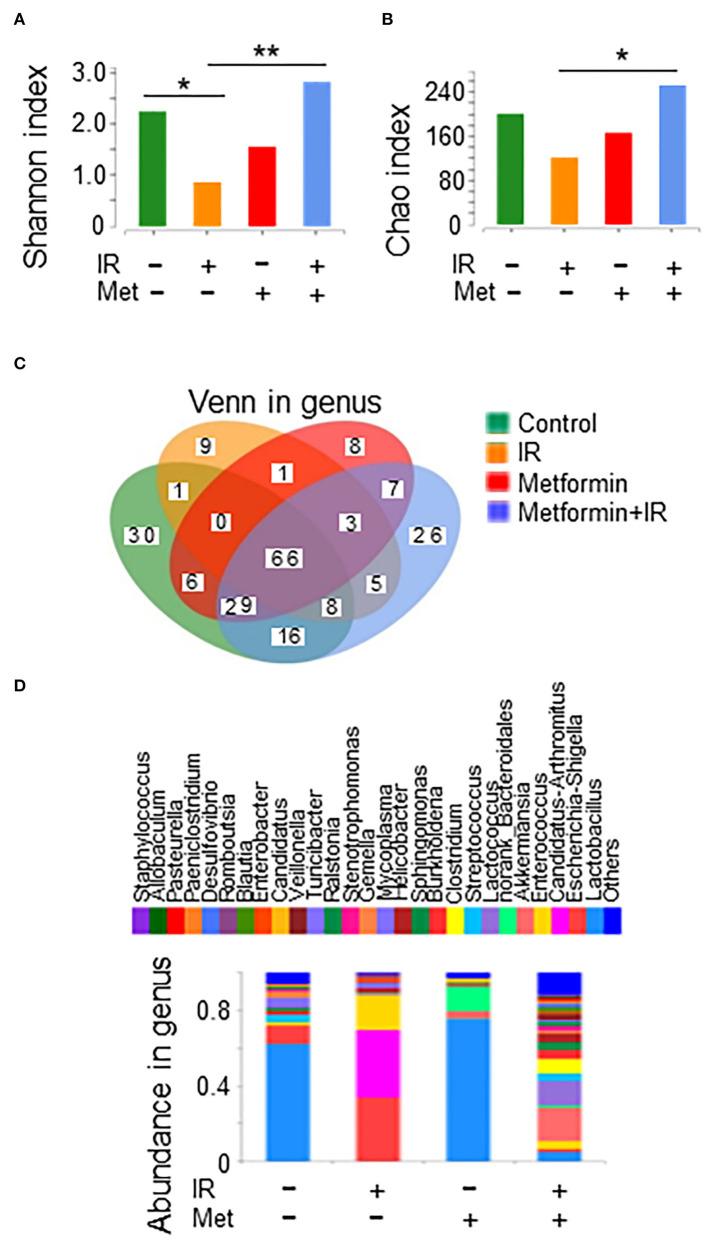
Metformin improves the composition and abundance of intestinal flora in abdominal IR mice. Female BALB/c mice were treated with abdominal IR and/or metformin as described in Figure 1. Total DNAs were isolated from intestinal contents as described in the Materials and methods section and subjected to 16S rRNA sequencing. Intestinal flora is shown as follows: **(A)** diversity (Shannon index), **(B)** abundance (Chao index), **(C)** Venn in genus level, and **(D)** bacterial composition in the intestine of mice treated with or without metformin. Data represent mean ± SEM. * *P* < 0.05, ** *P* < 0.01, *n* = 3, metformin+IR vs. IR. Also, see Supplementary Figures S2, S3.

It was reported that metformin, a first-line agent for the treatment of type 2 diabetes, improves upper small intestine microbiota composition and restores the SGLT1-dependent glucose sensing to regulate glucose homeostasis (Wu et al., 2017; Bauer et al., 2018). Thus, we asked whether metformin could improve dysbiosis in IR mice. We performed a 16S rRNA sequencing with ileal contents from IR mice treated with metformin. Evaluation of the credibility of the sequencing by Sobs curves and Shannon curves indicated that the number of sequencing samples was acceptable (Supplementary Figure S2). Treatment of metformin markedly reversed the reduction in gut microbiota diversity in IR mice in the Shannon index analysis (Figure 2A). The abundance of intestinal flora was also improved in IR mice treated with metformin in the Chao index analysis (Figure 2B). Collectively, our results suggest that metformin improved the diversity and abundance of intestinal flora in abdominal IR mice.

We further analyzed the influence of metformin on the composition of intestinal flora in mice. The Venn plot analysis, which is used to display the distribution of the number of elements between various sets, showed that there were 11 phyla and 75 genera in common between the IR and control mice, whereas metformin+IR and control mice shared 14 phyla and 119 genera (Supplementary Figure S3A; Figure 2C), indicating that treatment of metformin renders IR mice more similarities in intestinal flora composition as in non-irradiated healthy mice. The community composition analysis chart (bar chart) (Jain et al., 2021) showed that the dominant phylum in all mice was *Firmicutes*. The flora composition was similar. However, the relative abundance was different at the phylum and genus levels among control-, metformin-, and metformin + IR-treated mice. The composition of the IR group was quite different from that of the aforementioned three groups (Supplementary Figure S3B; Figure 2D). Based on the bacterial classification, the microbiota was divided into three distinct taxa in the sample hierarchical cluster map. The distance between metformin+IR and control mice was relatively close, indicating that the composition of the flora in the two groups was similar, whereas a far distance between IR and control group was observed, indicating less similarity in the composition of microbiota in the two groups (Supplementary Figure S3C). Similar results for the beneficial effects of metformin in the maintenance of microbiota composition in mice with or without IR were observed in the PCA analysis (Supplementary Figure S3D). Collectively, these results demonstrate that IR led to the disorder of intestinal flora composition in mice, which could be corrected by metformin.

### Metformin reduces IR-induced intestinal damage in mice by improving the composition and abundance of microbiota

**Figure 3:**
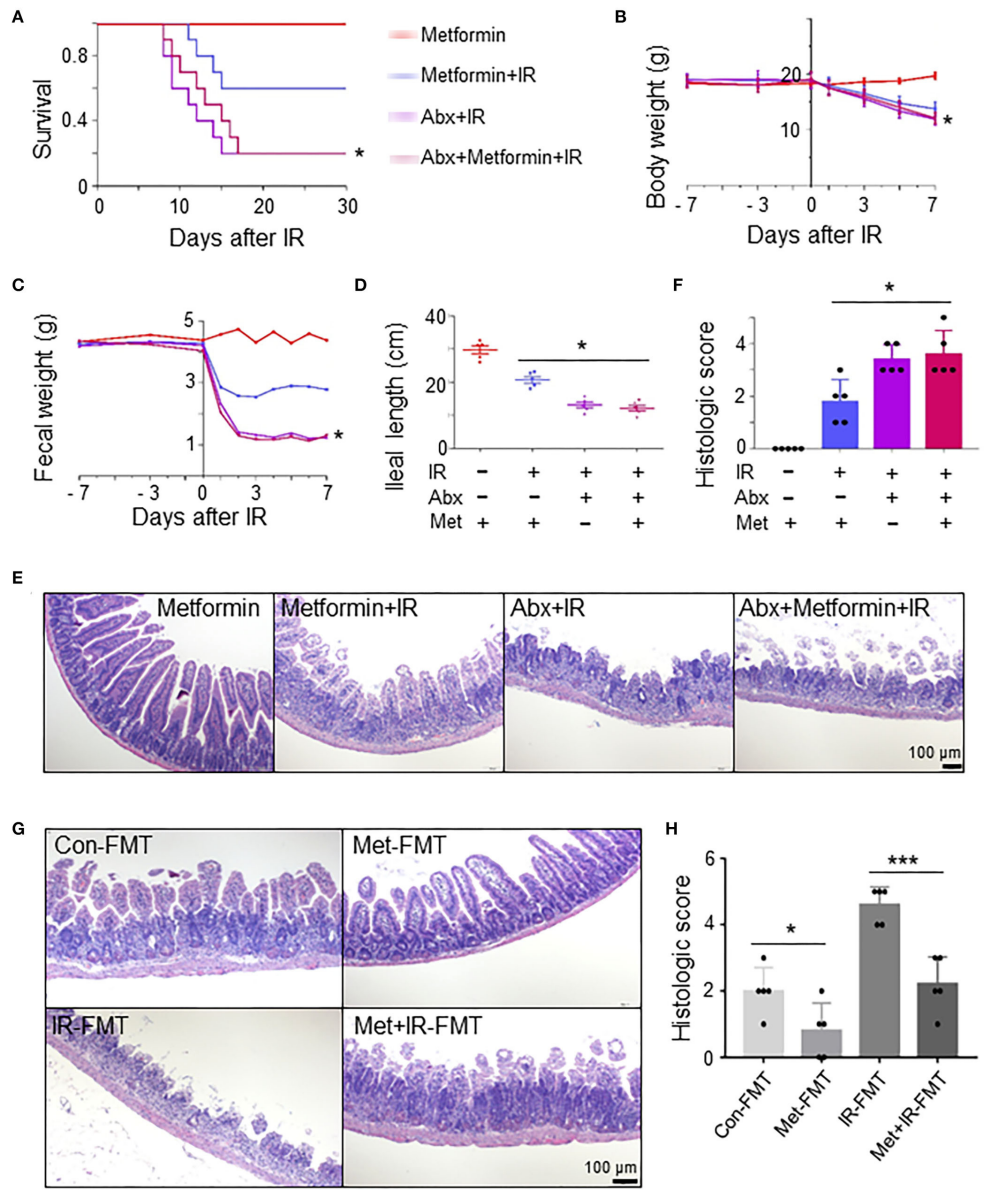
Metformin reduces IR-induced intestinal damage in mice by improving the composition and abundance of microbiota. Mice were pretreated with an antibiotic mixture for 4 weeks before metformin treatment. Mice were irradiated as described in Figure 1. Ilea were collected for light microscopy 3 days after IR. The mice were monitored for **(A)** survival, **(B)** body weight, **(C)** fecal output, **(D)** ileal length, **(E)** H&E staining of ileum, and **(F)** damage score of ileal tissue. * *P* < 0.05, Abx+metformin+IR vs. metformin+IR. **(G,H)** A total of 200 μl FMT using feces from control-, metformin-, IR-, or metformin+IR-treated mice were intragastrically administered into microbiota-eliminated mice (Abx) for 4 weeks continuously before and 1 week after IR to reorganize the intestinal microenvironment. **(G)** H&E staining of ileal tissues from IR mice received FMT with feces from control (Con-FMT), metformin-treated (Met-FMT), IR mice (IR-FMT), or metformin-treated IR mice (Met+IR-FMT). **(H)** Damage score of ileal tissues of mice in **(G)**. Data represent mean ± SEM. * *P* < 0.05, *** *P* < 0.001, *n* = 5.

To determine whether the role of metformin in radioprotection of the intestine is mediated by gut microbiota, we treated normal and microbiota-eliminated (Abx) mice with metformin followed by IR. Interestingly, metformin no longer bore a radioprotective effect on the intestine in the Abx mice. The survival and body weight were similar in Abx+IR mice with or without metformin treatment, which was significantly lower than those in control mice treated with metformin and IR (metformin+IR) (Figures 3A,B). The amount of feces was reduced, and ileal length was decreased in Abx mice with IR regardless of the application of metformin (Figures 3C,D). Histologically, the protective effects of metformin on intestinal epithelial cells in IR mice were abrogated in Abx mice (Figures 3E,F). These results indicate that intestinal flora is required for metformin-mediated alleviation of radioactive intestinal injury. To verify this hypothesis, we reconstituted the gut microbiome with feces from control or metformin-treated non-IR mice into gut microbiota-eliminated mice. Fecal microbiota transplant (FMT) from control or metformin-treated mice conferred microbiota-eliminated mice resistance to abdominal IR, and FMT from metformin-treated mice was more effective (Figures 3G,H). In addition, we also reconstituted the gut microbiome with feces from IR or metformin+IR mice into mice with intestinal flora elimination. Microbiota-eliminated mice transplanted with feces from IR mice are sensitive to abdominal IR. Intestinal epithelial damage and the histological score of the damaged intestine were severe (Figures 3G,H). However, feces from metformin+IR mice markedly reduced radiation-induced intestinal damage in the recipient mice (Figures 3G,H). Collectively, our data suggest that metformin reduces IR-induced intestinal damage in the presence of microbiota in mice.

### Metformin treatment increases the abundance of *Lactobacillus* in the intestine of mice

**Figure 4:**
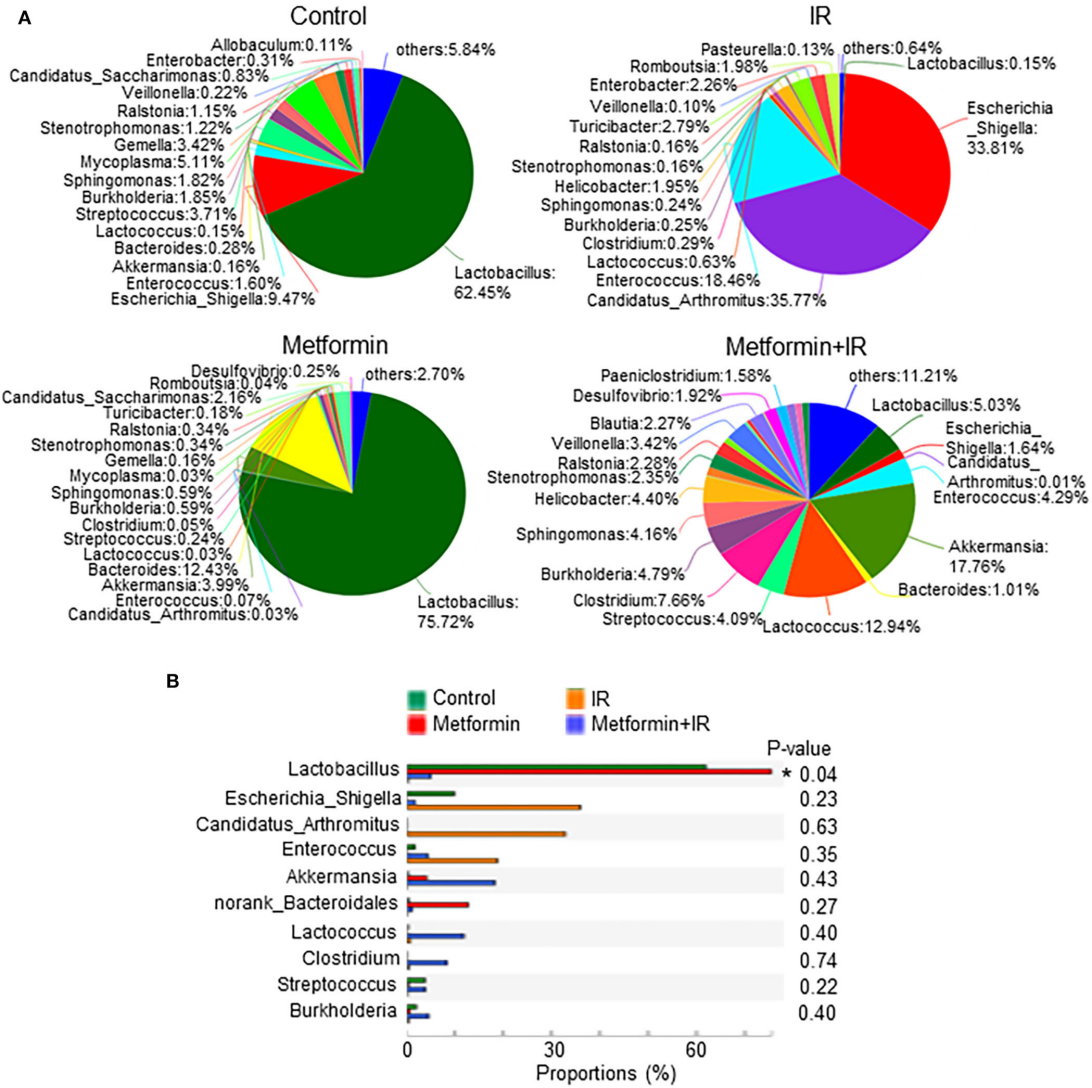
Metformin increases the abundance of *Lactobacillus*. Female BALB/c mice were treated with abdominal IR and/or metformin as described in Figure 1. Total DNAs were isolated from intestinal contents as described in the “Materials and methods” section and subjected to 16S rRNA sequencing. **(A)** Pie analysis and **(B)** comparison of bacteria in intestinal flora in mice treated with or without metformin and identification of *Lactobacillus* as the most changed bacteria in abundance after metformin treatment. Data represent mean ± SEM. * *P* < 0.05, *n* = 3, metformin+IR vs. IR.

We analyzed the species of intestinal flora in IR mice treated with metformin based on the Pie and heat maps. They showed that the dominant genus in control- and metformin-treated mice was *Lactobacillus* (Figure 4). The abundance of *Lactobacillus* and *Akkermansia* in metformin-treated mice was increased, whereas that of *Escherichia-Shigella* was decreased, as compared with those in control mice (Figure 4). In addition to the enrichment of *Lactobacillus* and *Akkermansia*, metformin-treated IR mice also displayed a striking increase in *Lactococcus, Streptococcus, Clostridium*, and *Burkholderia-Paraburkholderia* and a marked decrease in *Escherichia-Shigella, Candidatus-Arthromitus*, and *Enterococcus* (Figure 4A; Supplementary Figure S3E). Among them, *Lactobacillus* was the top genus identified in the increase due to exposure to metformin (Figure 4B).

### Metformin protects the murine intestine against IR in the presence of *Lactobacillus*

**Figure 5:**
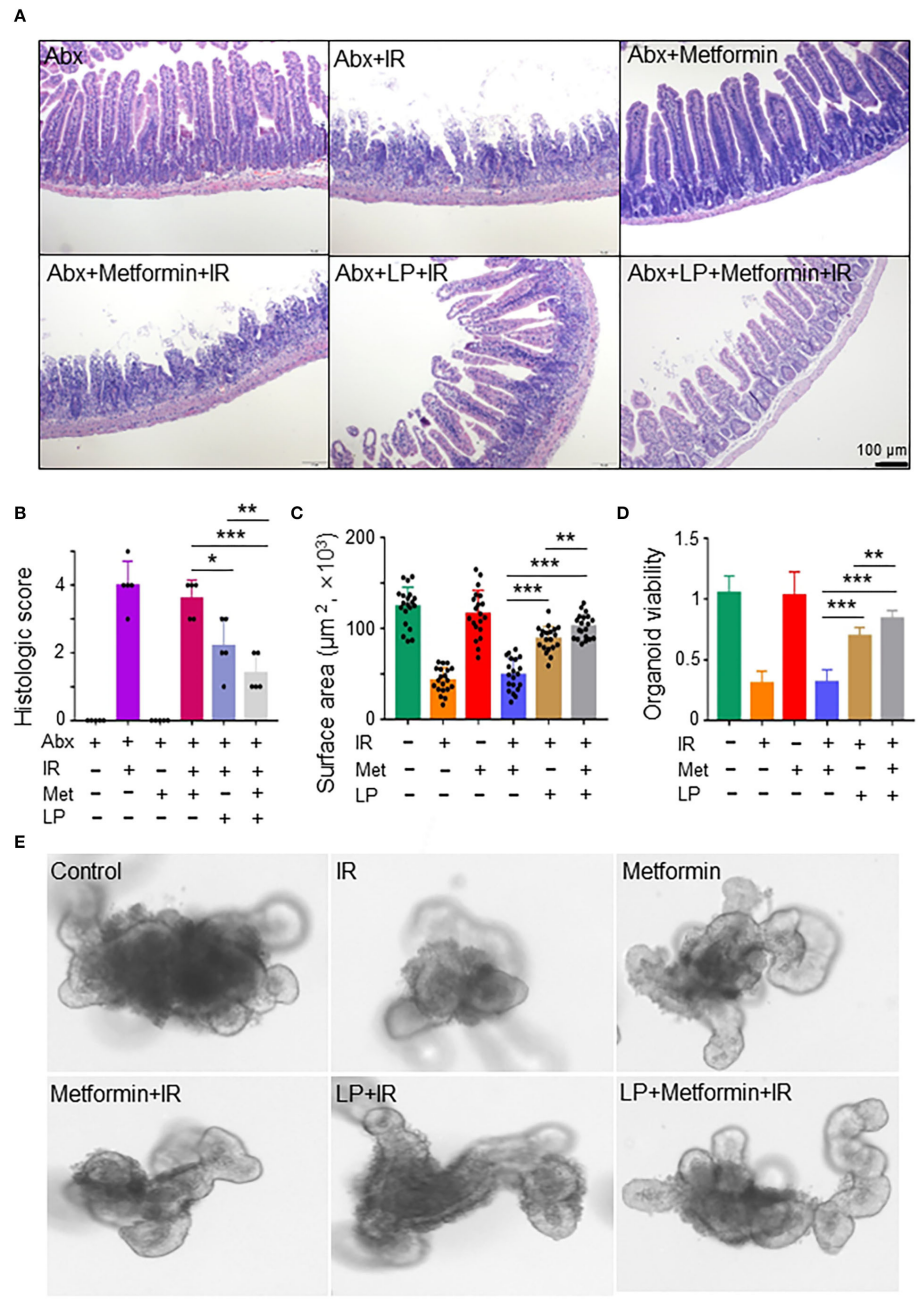
Metformin protects the murine intestine against IR in the presence of *Lactobacillus*. **(A,B)** Mice were pretreated with a mixture of antibiotics for 4 weeks before metformin and/or *Lactobacillus* treatment. Mice were irradiated as described in Figure 1. Ilea were collected for light microscopy 3 days after IR. **(A)** H&E staining of ilea. **(B)** Damage score of ileal tissues. Data represent mean ± SEM. * *P* < 0.05, *** *P* < 0.001, *n* = 5, compared with Abx+metformin+IR. **(C–E)** Ileal tissues of mice were collected for the intestinal organoid culture. Intestinal organoids were treated with metformin and/or *Lactobacillus* as described in the Materials and methods section. **(C)** Intestinal organoid surface area. **(D)** Intestinal organoid viability (OD_490nm_). **(E)** Representative intestinal organoid image. Scale bar = 20 μm. Data represent mean ± SEM. * *P* < 0.05, ** *P* < 0.01, *** *P* < 0.001, *n* = 5, compared with metformin+IR.

To characterize the role of *Lactobacillus* in metformin-induced radioprotection in abdominal IR mice, we administrated metformin into Abx mice by gavage. The administration of metformin did not protect against IR-induced intestinal damage in Abx mice (Figures 5A,B). Then we reconstituted Abx mice with *Lactobacillus* before metformin and IR treatment. Surprisingly, administration of *Lactobacillus* substantially lessened IR-induced intestinal damage in Abx mice (Figures 5A,B). More importantly, administration of *Lactobacillus* restored the radioprotective effect of metformin on the Abx mice (Figures 5A,B), further supporting the notion that metformin protects mice from IR-induced gut injury by improving the abundance of *Lactobacillus* in the intestine.

To further validate our finding that metformin reduces IR-induced intestinal damage by increasing the abundance of *Lactobacillus*, we analyzed the response of murine intestinal organoids to IR in the presence or absence of metformin and/or *Lactobacillus*. We found that IR inhibited intestinal organoid formation, and metformin alone had no protective effect on intestinal organoids treated with radiation (Figures 5C–E). However, combined treatment of metformin and *Lactobacillus* or *Lactobacillus* alone had an obviously protective effect on intestinal organoid formation following IR, leading to significantly larger organoids as compared with those treated with IR or metformin+IR (Figures 5C,E). In addition, intestinal organoids treated with metformin and *Lactobacillus* or *Lactobacillus* alone survived much better than those treated with PBS (control) or metformin alone under IR (Figures 5D,E). Together, these results support our notion that metformin exerts a radioprotective effect on murine intestines through *Lactobacillus*.

### Metformin enhances the intestinal barrier *via* activating the FXR signaling

**Figure 6:**
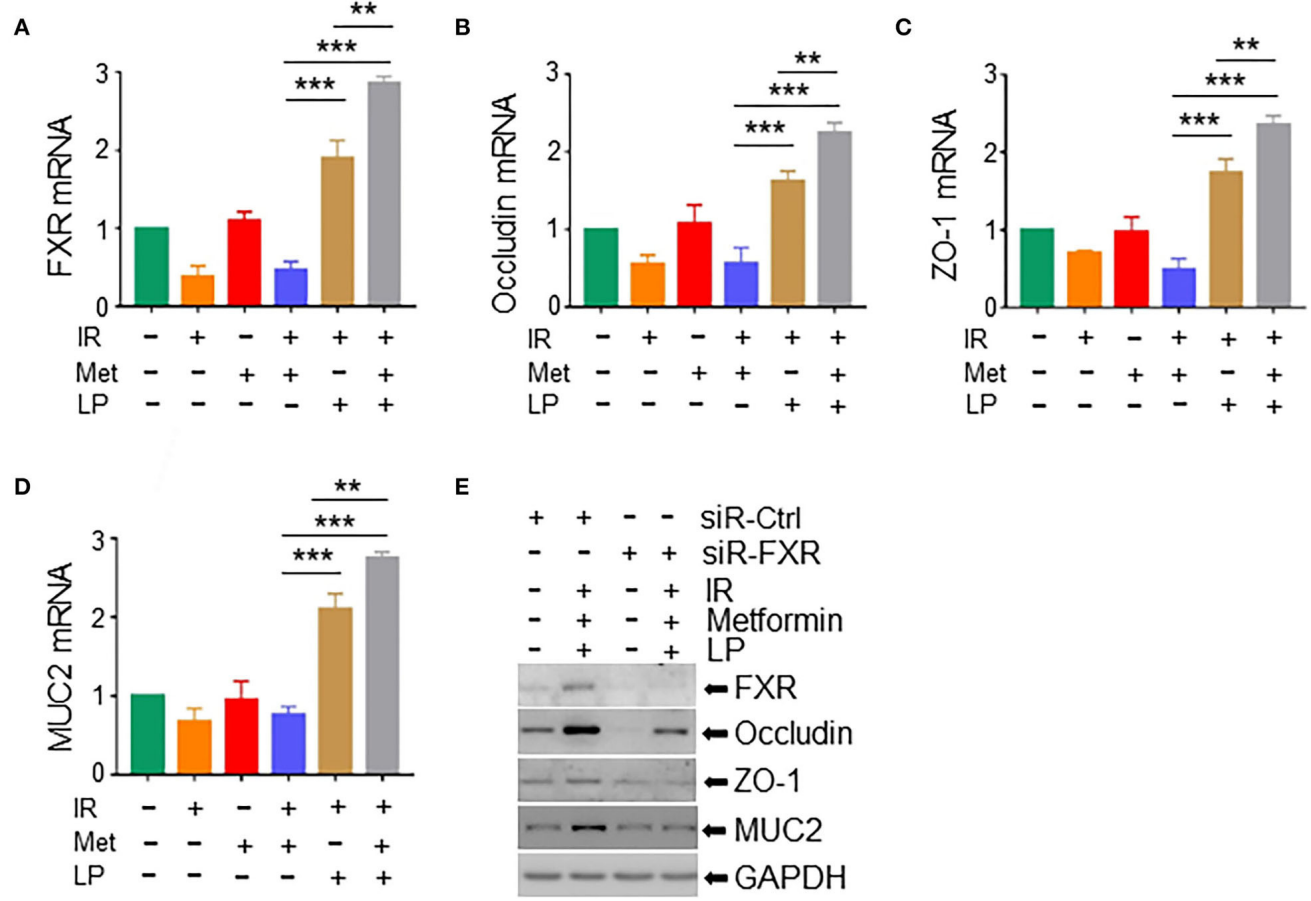
Metformin/*Lactobacillus* exerts a radioprotective effect *via* activating the FXR signaling. Intestinal organoids were treated with metformin and/or *Lactobacillus* as described in the Materials and methods section and Figure 5. Organoids were irradiated with 8-Gy X-ray. Total RNAs or whole cell lysates were extracted from the intestinal organoids 16 h after the irradiation. mRNA levels of FXR **(A)**, occludin **(B)**, ZO-1 **(C)**, and MUC2 **(D)** in intestinal organoids. qPCR was used for the quantitation. **(E)** Expressions of FXR, occludin, ZO-1, and MUC2 in intestinal organoids with FXR knockdown were detected with Western blotting. Data represent mean ± SEM. ** *P* < 0.01, *** *P* < 0.001, *n* = 5, compared with metformin+IR.

Studies have shown that gut microbiota decreased hepatic bile acid by activating farnesoid X receptor (FXR) in intestinal epithelial cells, leading to the alleviation of excessive hepatic bile-induced liver injury and fibrosis in mice (Liu et al., 2020). FXR activation promotes the expression of tight junction proteins to improve ileal barrier function (Verbeke et al., 2015), demonstrating a crucially protective role of FXR in the intestine. To determine the role of FXR in metformin-induced radioprotection, we analyzed the expression of FXR in irradiated intestinal organoids treated with metformin and/or *Lactobacillus*. We found that metformin substantially upregulated the level of FXR mRNA in irradiated mouse organoids in the presence of *LP*. Metformin-induced elevation of FXR was abrogated in irradiated murine intestinal organoids without the cotreatment of *LP* (Figure 6A). In addition, the mRNA levels of occludin and ZO-1, two important components of tight junctions between intestinal epithelial cells and MUC2, a mucus layer protein secreted by intestinal goblet cells, were also increased in irradiated mouse intestinal organoids pretreated with metformin and *LP*, indicating that cotreatment of metformin with *LP* increased the intestinal barrier function (Figures 6B–D). Consistently, knocking down of FXR markedly reduced the expression of occludin, ZO-1, and MUC2 induced by metformin and *LP* in mouse intestinal organoids (Figure 6E). Together, our data indicate that the activation of FXR signaling likely explains the radioprotective effect of metformin in mouse intestinal organoids.

### Activation of FXR reduces radiation-induced intestinal injury in microbiota-eliminated mice

**Figure 7:**
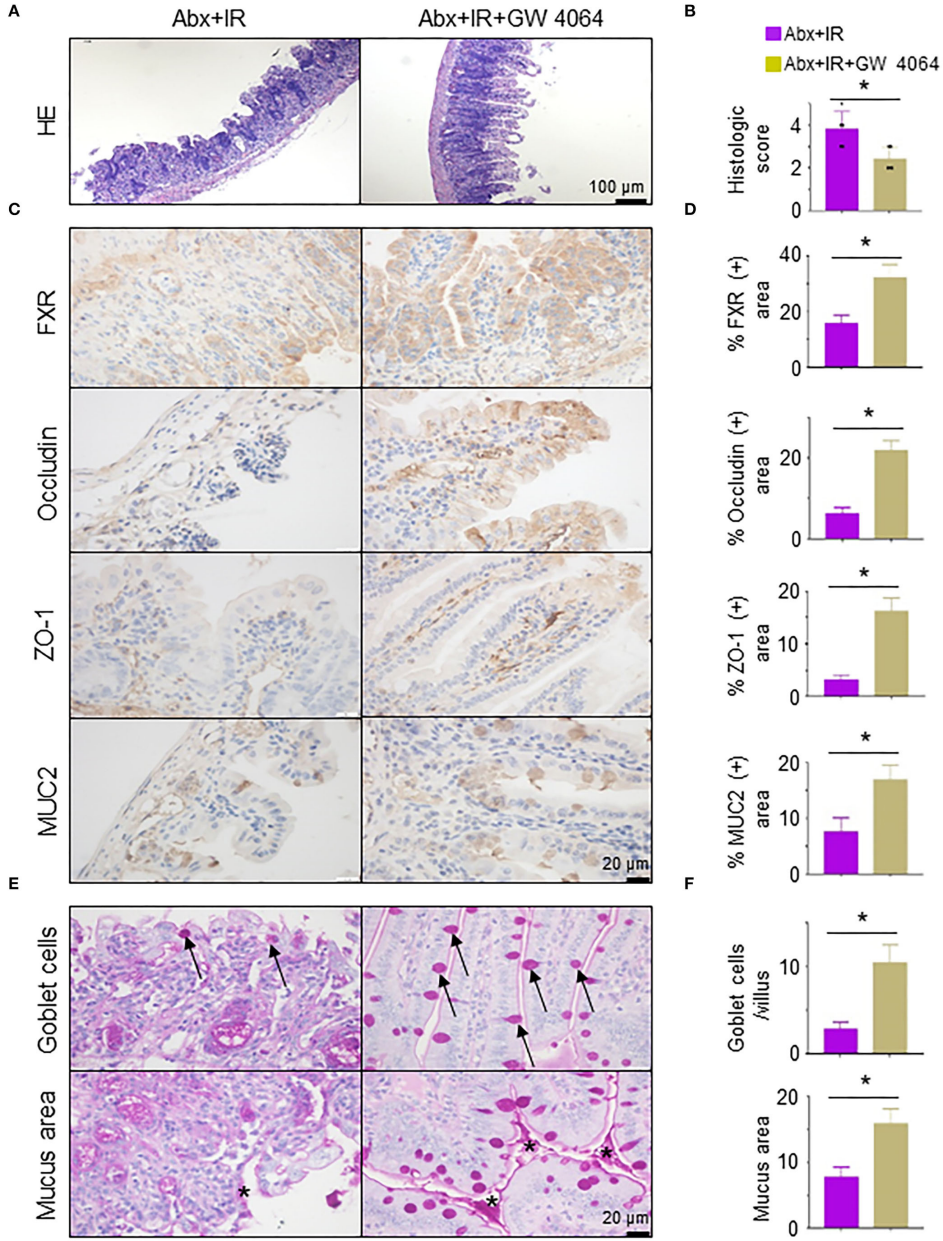
Activation of FXR reduces radiation-induced intestinal injury in microbiota-eliminated mice. Microbiota-eliminated mice (Abx) were injected intraperitoneally with an FXR activator (GW 4064, 30 mg/kg/day, MedChemExpress, China) 3 days before IR. Irradiation of the mice was performed as described in Figure 1. The mice were subjected to the following analyses. **(A)** H&E staining of ilea in IR mice treated with FXR activator GW 4064. **(B)** Damage score of ilea in **(A)**. **(C,D)** IHC staining was performed in ilea from IR mice treated with or without FXR activator GW 4064 **(C)**. Percentage of FXR, occludin, ZO-1, and MUC2 positive area in IHC staining of ilea in IR mice treated with FXR activator GW 4064 **(D)**. **(E,F)** PAS staining of goblet cells and mucus in the intestine of mice treated with FXR activator GW 4064. Arrows, goblet cells; Stars, mucus. **(F)** Goblet cells per villus and quantitation of the mucus area. Data represent mean ± SEM. * *P* < 0.05, *n* = 5, Abx+GW 4064+IR vs. Abx+IR.

To validate the necessity of FXR signaling activation in metformin/*Lactobacillus-*mediated radioprotective effects on intestinal epithelia, we pretreated abdominal IR mice with GW 4064, an FXR activator (Seok et al., 2014). The application of FXR activator GW 4064 markedly alleviated intestinal tissue damage in microbiota-eliminated IR mice (Figures 7A,B). To determine how activation of FXR signaling reduces intestinal damage by IR, we detected intestinal epithelial barrier integrity in abdominal IR mice treated with or without GW 4064. Expressions of FXR, occludin, ZO-1, and MUC2 in the intestine of abdominal IR mice treated with GW 4064 were increased as compared with those in IR mice without treatment with GW 4064 (Figures 7C,D). More importantly, the number of goblet cells in the small intestine of IR mice was upregulated after GW 4064 treatment (Figure 7E, upper panels, Figure 7F). The mucus layer areas on the surface of the small intestinal villi were also increased (Figure 7E, lower panels, Figure 7F). Collectively, our data suggest that activation of FXR maintains the integrity of intestinal epithelial barriers and promotes the reduction of IR-induced intestinal injury.

### Inhibition of FXR abolishes the protective effect of *Lactobacillus*

To further validate the role of FXR activation in the radioprotection of metformin/*Lactobacillus* against IR-induced intestinal injury, we pretreated the mice with Gly-β-MCA, an FXR inhibitor (Fu et al., 2019), before abdominal IR. Surprisingly, exposure to the FXR inhibitor Gly-β-MCA abrogated *Lactobacillus*-mediated protection in the ileum in IR mice (Supplementary Figures S4A,B). Expression of FXR, occludin, ZO-1, and MUC2 was substantially reduced in the intestine of abdominal IR mice treated with Gly-β-MCA as compared with those in IR mice treated without Gly-β-MCA (Supplementary Figures S4C,D). In addition, the number of goblet cells and the area of the mucus layer were dramatically decreased in abdominal IR mice treated with Gly-β-MCA in spite of the existence of *LP* (Supplementary Figures S4E,F). Taken all together, our results suggest that metformin/*Lactobacillus-*activated FXR signaling upregulates the levels of tight junction proteins and mucins in intestinal epithelia, increases the number of goblet cells, and augments the mucus layer thickness to maintain the integrity of intestinal epithelial barrier (Supplementary Figure S5), which eventually contributes to reduced radiation intestinal injury.

### Metformin promotes the abundance of *Lactobacillus* in the intestine of abdominal IR patients

**Figure 8:**
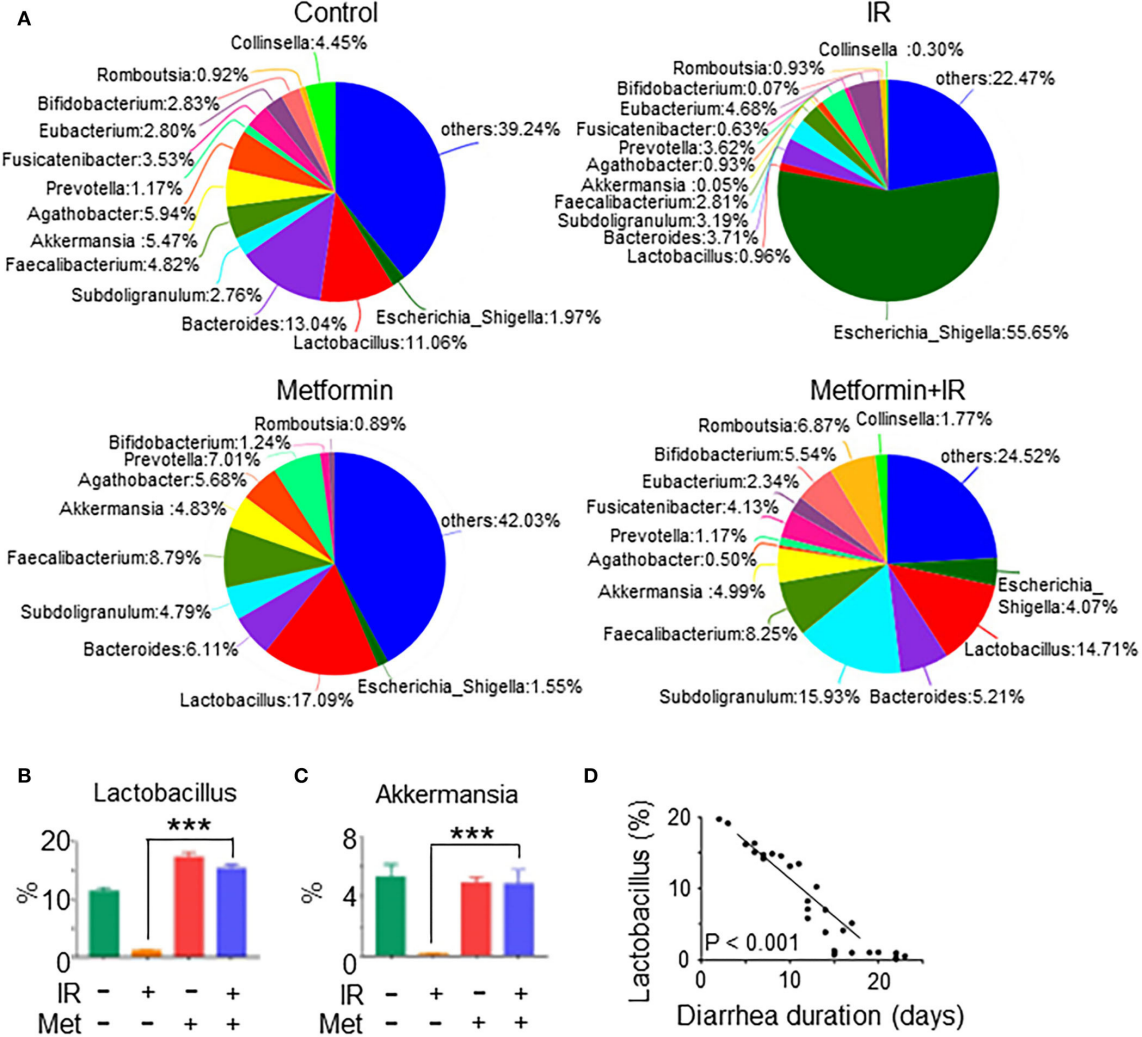
Metformin improves the composition and abundance of intestinal flora in abdominal IR patients. Feces samples from cervical cancer patients receiving abdominal IR with or without metformin treatment and healthy subjects were collected. Total DNAs were isolated from the feces as described in the Materials and methods section and subjected to 16S rRNA sequencing. **(A)** Pie analysis of intestinal flora in abdominal IR patients with or without metformin treatment and healthy subjects. **(B,C)** Percentage of *Lactobacillus* **(B)** and *Akkermansia* **(C)** in the intestinal flora. **(D)** Correlation of *Lactobacillus* abundance and diarrhea duration in abdominal IR patients. Data represent mean ± SEM. *** *P* < 0.001, compared with abdominal IR patients, *n* = 15 in each group.

To investigate the effect of metformin on gut microbiota composition and the prevention of diarrhea in patients with abdominal radiotherapy, we collected feces from abdominal IR patients with or without metformin treatment (500 mg, 3 times per day, oral administration for more than 3 months before radiotherapy) and age-matched healthy subjects (HC) for 16S rRNA sequencing. The sequencing results showed that the intestinal flora of IR patients was different from that of HC. Metformin treatment improved the composition of gut microbiota of IR patients (Figure 8A). To clarify the effect of metformin on gut microbiota composition, we performed a statistical analysis of specific microbiota. We found that the abundance of *Lactobacillus, Akkermansia, Bifidobacterium, Romboutsia, Subdoligranulum, Faecalibacterium*, and *Bacteroides* was increased, and *Escherichia-Shigella, Prevotella*, and *Agathobacter* decreased in the feces of IR patients treated with metformin as compared with those without metformin (Figures 8A–C), among which *Lactobacillus* raised the most in abundance (Figure 8B). The amount of *Lactobacillus* was negatively correlated with the diarrhea duration of patients (Figure 8D). Collectively, our data support the observation that abdominal irradiation leads to gut dysbiosis in patients. Metformin promotes the abundance of *Lactobacillus* in the intestine of abdominal IR patients.

## Discussion

Irradiation is one of the most widely used strategies in abdominal tumor treatment, but the toxicity of IR in normal tissues is usually dose-limiting, as it often leads to IR-induced intestinal injury. As a result, there is an increasing interest to identify agents that manage both short- and long-term radiation damage (Fransson and Widmark, 2007; De Ruysscher et al., 2019).

Metformin is the most widely used drug for the treatment of type 2 diabetes (García-Calzón et al., 2020; Koh et al., 2020). Metformin exerts its antihyperglycemic action mainly by reducing hepatic glucose production (Hunter et al., 2018), hence reducing the level of glucose. It was found that metformin also alleviates type 2 diabetes by maintaining the homeostasis of intestinal flora (Bauer et al., 2018). The role of metformin in IR-induced intestinal dysbiosis was not characterized previously. In this study, we identified that metformin improved the microbiota abundance and diversity in the intestine of IR mice (Figures 2A,B). In the follow-up analysis, we identified that *Lactobacillus* in intestinal flora was markedly elevated by metformin (Figure 4). Elimination of intestinal microbiota abrogated the radioprotective effect of metformin on the intestine of mice. In addition, we found that *Lactobacillus* abundance was significantly increased in the intestine of abdominal radiotherapy patients treated with metformin and its abundance was negatively correlated with the diarrhea duration (Figure 8). Thus, our data endorse a robust role of metformin in the radioprotection of the intestine by maintaining the integrity of intestinal microbiota.

Metformin was previously reported to enhance radiation sensitivity (Gulati et al., 2020) and improve radiation-induced lung injury, alleviate pulmonary fibrosis and inflammatory infiltration, and hence bear a potential for application in radiation protection (Wang et al., 2017). Chen et al. reported that metformin alleviates radiation-induced intestinal injury by optimizing mitophagy, an AMPK-dependent process (Chen et al., 2020). However, the impact of metformin on gut microbiota in radiation-induced intestinal injury is unclear. In view of the capability of metformin in the improvement of intestinal flora in type 2 diabetes, in this study, we asked whether metformin was able to enhance the composition and abundance of intestinal flora in IR mice, and hence alleviate the intestinal injury and improve the survival of the mice. We found that metformin improved the composition and abundance of intestinal microbiota, in particular the enrichment of *Lactobacillus* in the intestine of IR mice. Fecal microbiota transplant (FMT) from metformin-treated mice or administration of *Lactobacillus* conferred microbiota-eliminated mice resistance to abdominal IR and promoted the survival of mice. Furthermore, we identified that metformin enhanced the intestinal barrier integrity *via Lactobacillus-*mediated activation of the FXR signaling in intestinal epithelial cells. More importantly, in the microbiota-eliminated mice, metformin lacked the protective effect against abdominal IR, further supporting the notion that intestinal flora, especially *Lactobacillus*, played a crucial role in the radioprotective effect of metformin. These findings provide robust evidence warranting that metformin is a novel agent for the treatment of IR-induced intestinal injury. Metformin is also reported to be able to ameliorate IR-induced bone marrow injury (Xu et al., 2015). It is an interesting question to be explored in the future to determine whether metformin exerts a radioprotective effect on the bone marrow through the intestinal flora.

The pivotal contributor to IR-induced intestinal injury is increased intestinal permeability. Therefore, we focused on FXR, a bile acid-responsive nuclear transcription factor, which is essential for the regulation of hepatic bile acid and lipid and carbohydrate metabolism (Vanwijngaerden et al., 2011; Mudaliar et al., 2013; Verbeke et al., 2014). Interestingly, FXR is also highly expressed in the intestine (Figure 6; Supplementary Figure S4), indicating that the effect of FXR may extend beyond the liver. The crucial role of FXR in maintaining intestinal homeostasis and its involvement in the gut-liver axis are increasingly recognized. In cholestasis, activation of FXR improves ileal barrier function (Modica et al., 2012). Lack of FXR is associated with increased intestinal permeability of the gastrointestinal tract (Wang et al., 2008; Gadaleta et al., 2011). In this study, we observed that knockdown or suppression of FXR led to the reduction in occludin, ZO-1, and MUC2 in murine intestinal organoids (Figure 6) and depletion of radioprotective effect of metformin and *LP* on IR mice (Supplementary Figure S4). Collectively, our data suggest that the activation of FXR signaling is important for the radioprotective effect of metformin on mouse intestines.

In addition, we applied GW 4064, a synthetic FXR agonist, to mice and found that activation of FXR alleviated IR-induced intestinal damage and strengthened the intestinal barrier in microbiota-eliminated mice. In stark contrast, an opposite outcome was obtained in IR mice treated with FXR inhibitor Gly-β-MCA, bile acid, and potent, stable, intestine-selective, and high-affinity FXR inhibitor (Jiang et al., 2015). These data support the conclusion that activation of FXR signaling is essential for the alleviation of IR-induced intestinal damage.

Taken together, in this study, using the abdominal irradiation mouse model, we made a series of novel findings. We demonstrated that metformin improved the composition and abundance of intestinal microbiota, in particular the enrichment of *Lactobacillus* in the intestine of IR mice. FMT from metformin-treated mice or administration of *Lactobacillus* conferred microbiota-eliminated mice resistant to abdominal IR and promoted the survival of mice. Furthermore, we identified that metformin enhanced the intestinal barrier integrity *via Lactobacillus-*induced activation of the FXR signaling in intestinal epithelial cells. These findings provide robust evidence warranting that metformin is a novel agent for the treatment of IR-induced intestinal injury. Our findings not only provide new insights into the role of gut microbiota in radiation-induced intestinal injury but also shed new light on the application of probiotics for the protection of radiation-damaged individuals.

## Conclusion

Metformin/*Lactobacillus* activated the FXR signaling in intestinal epithelial cells and hence strengthened the intestinal barrier. Corrections of abnormal gut microbiota, such as administration of *Lactobacillus* or elevation of the probiotic by metformin, could be a novel therapeutic strategy for IR-induced intestinal damage.

## Data availability statement

The data presented in the study are deposited in the figshare.com repository, accession number 21164911.V1.

## Ethics statement

The animal study was reviewed and approved by the Institutional Animal Care and Use Committee (IACUC) at Henan University, China.

## Author contributions

Y-PJ designed the study and wrote the manuscript. J-YY, M-JL, LL, J-RG, K-YH, K-KW, C-YC, B-ZY, and D-DD performed the experiments. HZ, QD, G-LL, and J-HW explained and discussed the data. Z-XX contributed to the conception and writing. All authors read and approved the final manuscript.

## Funding

This study was supported by the National Natural Science Foundation of China (Nos. 82020108024, 32161143021, and 81772924).

## Conflict of interest

The authors declare that the research was conducted in the absence of any commercial or financial relationships that could be construed as a potential conflict of interest.

## Publisher's note

All claims expressed in this article are solely those of the authors and do not necessarily represent those of their affiliated organizations, or those of the publisher, the editors and the reviewers. Any product that may be evaluated in this article, or claim that may be made by its manufacturer, is not guaranteed or endorsed by the publisher.

## Supplementary material

Supplementary Figure S1 Metformin treatment does not affect blood glucose, drinking water, and food intake in IR mice. Healthy BALB/c mice raised in an SPF environment were irradiated with 8 Gy abdominal IR (X-ray, dosage rate 1 Gy/min) or mock treatment. Metformin was intragastrically administered to the mice at a dose of 250 mg/kg/day for 7 days before and 3 days after IR for a total of 10 days. The mice were subjected to the following analyses. **(A)** Blood glucose, **(B)** drinking water intake, **(C)** food intake, **(D)** damage score of ileal tissue, and **(E)** intestinal microvillus length (μm) of ilea. Data represent mean ± SEM. ^***^ *P* < 0.001, *n* = 5, NS represents no statistical difference, compared with IR.

Supplementary Figure S2 16S rRNA sequencing of murine ileal contents after radiation. Healthy BALB/c mice raised in an SPF environment were irradiated with 8 Gy abdominal IR (X-ray, dosage rate 1 Gy/min) or mock treatment. Intestinal contents around the ileum of the mice were collected 3 days after the IR. Total DNAs were isolated from the contents as described in the Materials and methods section and subjected to 16S rRNA sequencing. **(A)** Sobs index dilution curves of intestinal flora. **(B)** Shannon index dilution curves of intestinal flora.

Supplementary Figure S3 Metformin treatment improves gut microbiota composition. Mice were treated and irradiated as described in Supplementary Figure S1. Intestinal contents around the ileum of mice were collected 3 days after IR. Total DNAs were isolated from the contents as described in the “Materials and methods” section and subjected to 16S rRNA sequencing. Intestinal flora is shown as follows. **(A)** Venn on phylum level, **(B)** composition on phylum level, **(C)** hierarchical clustering maps, **(D)** PCA (principal component analysis), and **(E)** heat map. Data represent mean ± SEM. *n* = 3, metformin+IR vs. IR.

Supplementary Figure S4 Inhibition of FXR abolishes the radioprotective effect of *Lactobacillus*. Microbiota-eliminated (Abx) mice reconstituted with *Lactobacillus* were injected intraperitoneally with FXR inhibitor Gly-β-MCA (30 mg/kg/day, MedChemExpress, China). Irradiation of mice was performed 3 days after the treatment, as described in Figure 1. Mice were subjected to the following analyses. **(A)** H&E staining of ilea in IR mice treated with FXR inhibitor Gly-β-MCA. **(B)** Damage score of ilea in IR mice treated with or without FXR inhibitor Gly-β-MCA in **(A)**. **(C,D)** IHC staining **(C)** and its quantitation **(D)** were performed in ilea from IR mice treated with FXR inhibitor Gly-β-MCA. **(E,F)** PAS staining of goblet cells and mucus in the intestine of IR mice treated with FXR inhibitor Gly-β-MCA. Arrows, goblet cells; Stars, mucus. **(F)** Goblet cells per villus and quantitation of the mucus area. Data represent mean ± SEM. ^*^ *P* < 0.05, *n* = 5, Abx + LP + MCA + IR vs. Abx + LP + IR.

Supplementary Figure S5 The role of metformin/*Lactobacillus-*FXR signaling in the reduction of radiation-induced intestinal injury. Metformin activates FXR signaling by increasing the abundance of *Lactobacillus* to upregulate levels of tight junction proteins and mucins in intestinal epithelia, increase the number of goblet cells, and augment the mucus layer thickness to maintain the integrity of the intestinal epithelial barrier in abdominal IR subjects.

## Abbreviations

IR: irradiation
Abx: elimination of intestinal microbiota through the antibiotic mixture
FMT: fecal microbiota transplantation
FXR: farnesoid X receptor
SGLT1: sodium-glucose cotransporter-1
IHC: immunohistochemistry
HE: hematoxylin-eosin
IECs: intestinal epithelial cells
MOI: multiplicity of infection
*LP*: *Lactobacillus plantarum*
PAS: periodic acid-Schiff.
