# Supplementary figures and images for: Metformin alleviates irradiation-induced intestinal injury by activation of FXR in intestinal epithelia
Source: Front Microbiol. 2022 Oct 13;13:932294. doi: 10.3389/fmicb.2022.932294 (PMC9608595; doi:10.3389/fmicb.2022.932294)

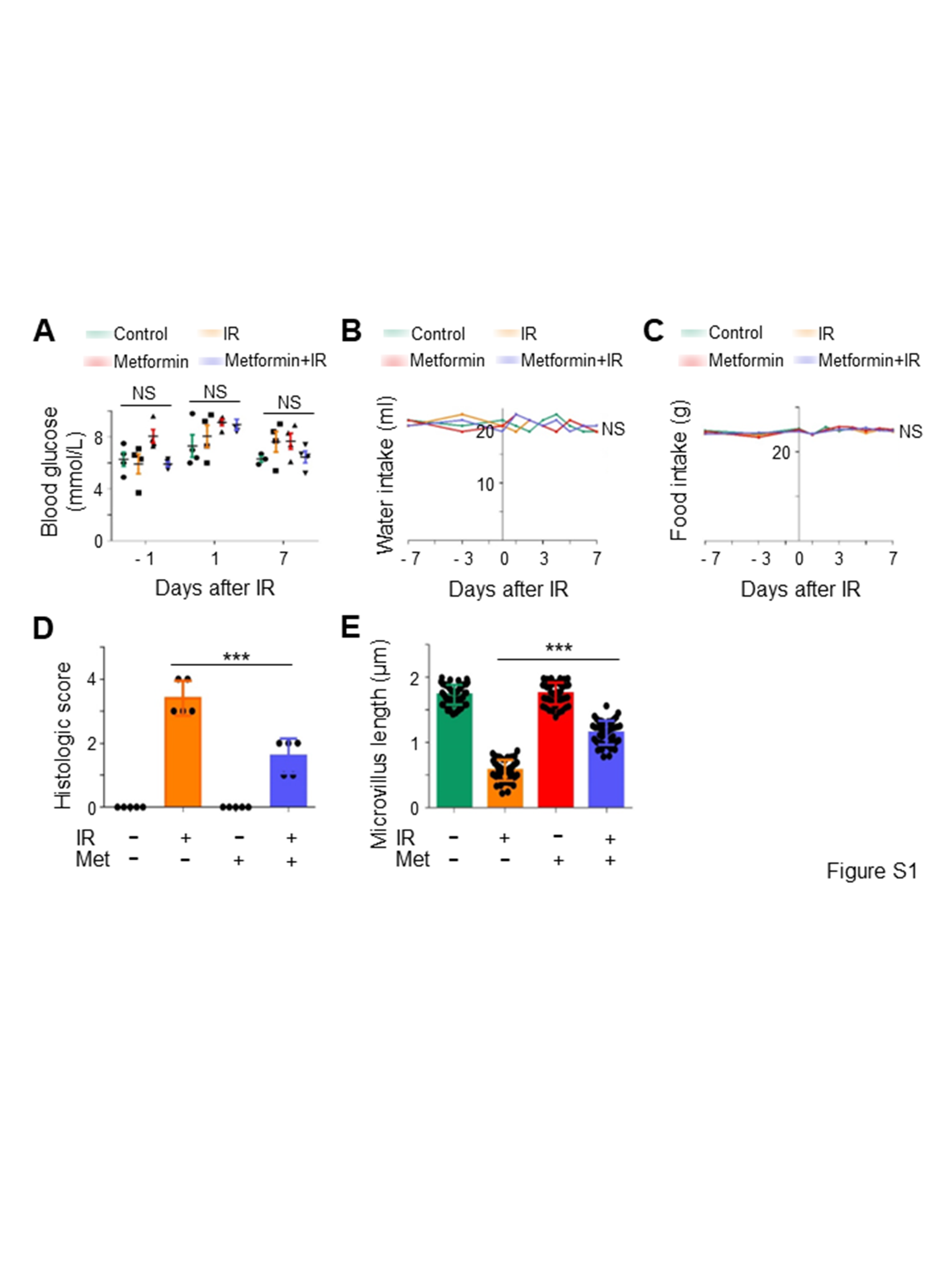

Supplement: Supplementary Figure S1 — Metformin treatment does not affect blood glucose, drinking water, and food intake in IR mice. Healthy BALB/c mice raised in an SPF environment were irradiated with 8 Gy abdominal IR (X-ray, dosage rate 1 Gy/min) or mock treatment. Metformin was intragastrically administered to the mice at a dose of 250 mg/kg/day for 7 days before and 3 days after IR for a total of 10 days. The mice were subjected to the following analyses. (A) Blood glucose, (B) drinking water intake, (C) food intake, (D) damage score of ileal tissue, and (E) intestinal microvillus length (μm) of ilea. Data represent mean ± SEM. *** P < 0.001, n = 5, NS represents no statistical difference, compared with IR. [file Image_1.TIF]

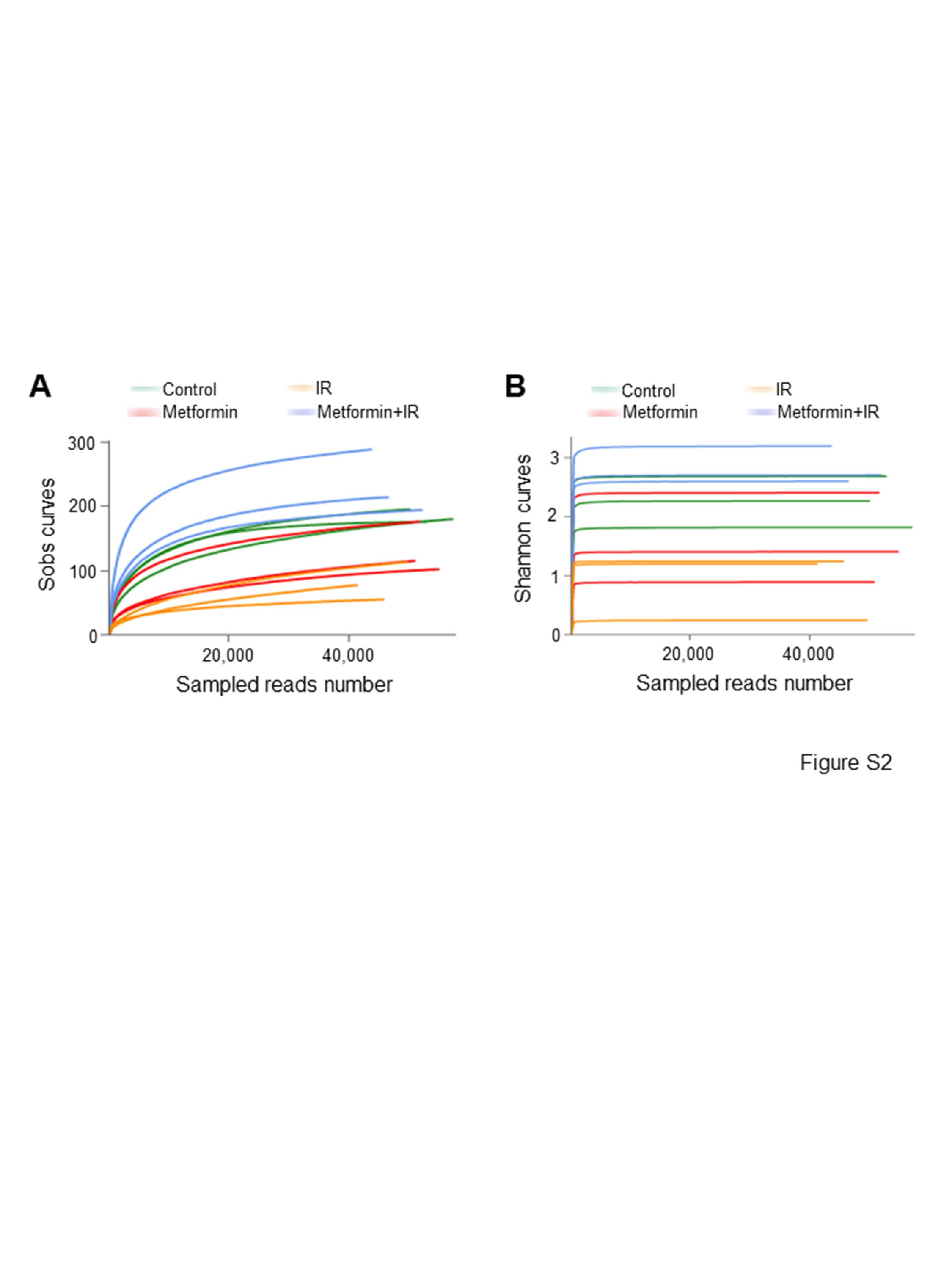

Supplement: Supplementary Figure S2 — 16S rRNA sequencing of murine ileal contents after radiation. Healthy BALB/c mice raised in an SPF environment were irradiated with 8 Gy abdominal IR (X-ray, dosage rate 1 Gy/min) or mock treatment. Intestinal contents around the ileum of the mice were collected 3 days after the IR. Total DNAs were isolated from the contents as described in the Materials and methods section and subjected to 16S rRNA sequencing. (A) Sobs index dilution curves of intestinal flora. (B) Shannon index dilution curves of intestinal flora. [file Image_2.TIF]

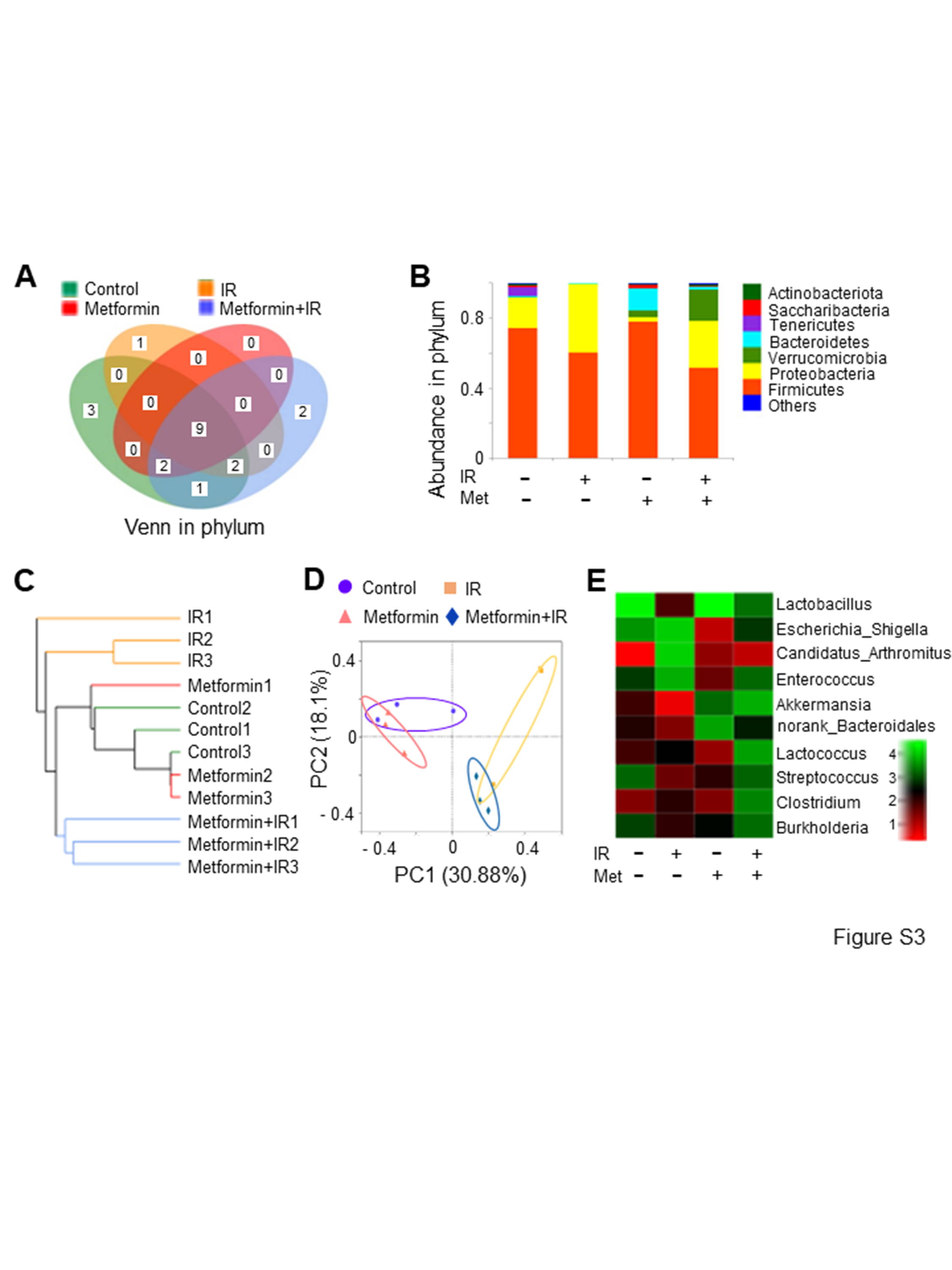

Supplement: Supplementary Figure S3 — Metformin treatment improves gut microbiota composition. Mice were treated and irradiated as described in Supplementary Figure S1. Intestinal contents around the ileum of mice were collected 3 days after IR. Total DNAs were isolated from the contents as described in the “Materials and methods” section and subjected to 16S rRNA sequencing. Intestinal flora is shown as follows. (A) Venn on phylum level, (B) composition on phylum level, (C) hierarchical clustering maps, (D) PCA (principal component analysis), and (E) heat map. Data represent mean ± SEM. n = 3, metformin+IR vs. IR. [file Image_3.TIF]

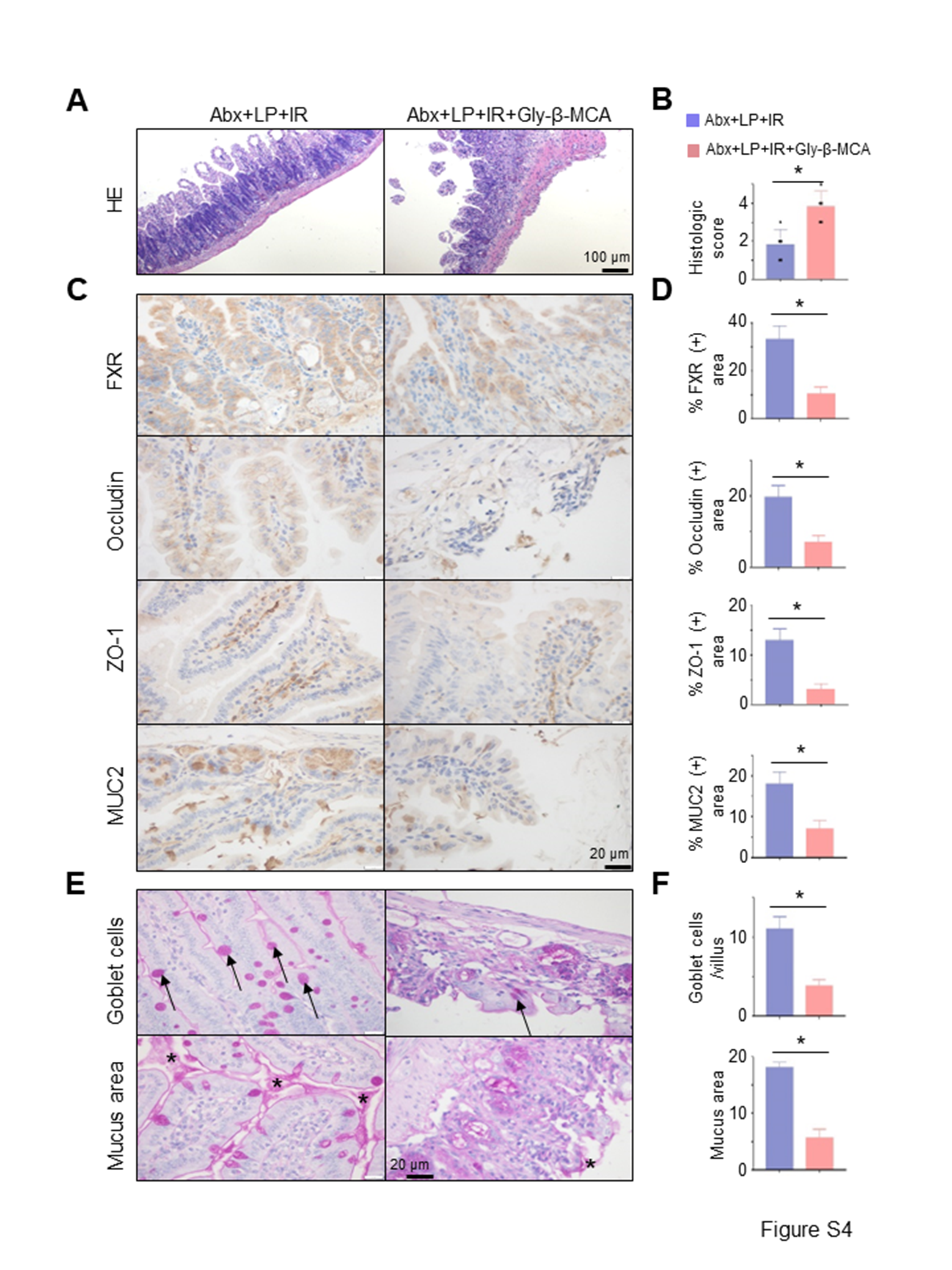

Supplement: Supplementary Figure S4 — Inhibition of FXR abolishes the radioprotective effect of Lactobacillus. Microbiota-eliminated (Abx) mice reconstituted with Lactobacillus were injected intraperitoneally with FXR inhibitor Gly-β-MCA (30 mg/kg/day, MedChemExpress, China). Irradiation of mice was performed 3 days after the treatment, as described in Figure 1. Mice were subjected to the following analyses. (A) H&E staining of ilea in IR mice treated with FXR inhibitor Gly-β-MCA. (B) Damage score of ilea in IR mice treated with or without FXR inhibitor Gly-β-MCA in (A). (C,D) IHC staining (C) and its quantitation (D) were performed in ilea from IR mice treated with FXR inhibitor Gly-β-MCA. (E,F) PAS staining of goblet cells and mucus in the intestine of IR mice treated with FXR inhibitor Gly-β-MCA. Arrows, goblet cells; Stars, mucus. (F) Goblet cells per villus and quantitation of the mucus area. Data represent mean ± SEM. * P < 0.05, n = 5, Abx + LP + MCA + IR vs. Abx + LP + IR. [file Image_4.TIF]

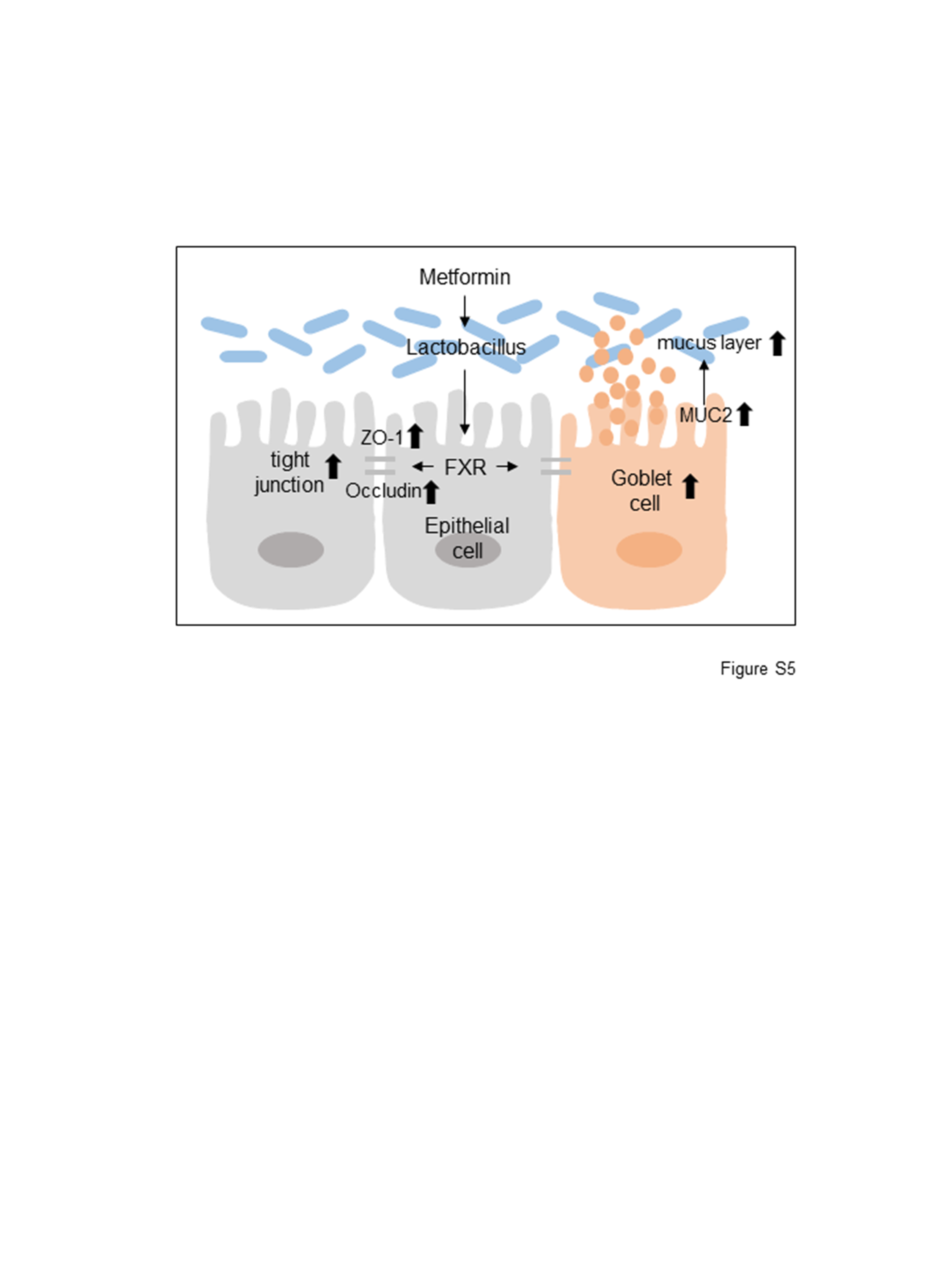

Supplement: Supplementary Figure S5 — The role of metformin/Lactobacillus-FXR signaling in the reduction of radiation-induced intestinal injury. Metformin activates FXR signaling by increasing the abundance of Lactobacillus to upregulate levels of tight junction proteins and mucins in intestinal epithelia, increase the number of goblet cells, and augment the mucus layer thickness to maintain the integrity of the intestinal epithelial barrier in abdominal IR subjects. [file Image_5.TIF]
